# An unexpected cause of chest pain

**DOI:** 10.11604/pamj.2024.47.28.42634

**Published:** 2024-01-23

**Authors:** Yoen Young Chuah, Yi-Chun Chan

**Affiliations:** 1 Division of Gastroenterology and Hepatology, Department of Internal Medicine, Pingtung Christian Hospital, Pingtung, Taiwan,; 2 Department of Nursing, Meiho University, Pingtung, Taiwan

**Keywords:** Toothbrush, chest pain, endoscopy, esophageal ulcer

## Image in medicine

A 44-year-old woman presented initially at the outpatient clinic with persistent chest pain since morning. She claimed to have swallowed a toothbrush accidentally while brushing her teeth. There was no history of psychiatric disorders including bulimia. A subsequent chest X-ray did not show any radio-opaque object in her esophagus (A). The patient was deferred from further management at a previous emergency department visit due to a lack of X-ray evidence of radio-opaque impaction. Emergent upper endoscopy 7 hours after the incident was performed that found the stalk of the impacted toothbrush at the lower esophagus with superficial ulcerations beneath the stalk (B, with arrows). With a polypectomy snare, the 18 cm toothbrush was successfully removed without immediate complication (C). A proton pump inhibitor was prescribed. In the absence of typical X-ray findings (especially in a child), history and clinical suspicion would be important. Accidental ingestion of toothbrushes is rare. For unknown reasons, young females (mean age was 27.6 years) were more likely to ingest toothbrushes by accident. The majority of reported cases were due to vomiting induction (38%, 19/50) and only 10% (9/50) were due to teeth brushing (our case). Ingested toothbrushes can be easily identified on X-ray if with radiopaque wires in nylon bristles but not all toothbrushes have metallic elements as in our case. Impacted toothbrushes can be removed successfully with endoscopy, spontaneous passage through the rectum does not occur, and surgical removal is rarely needed.

**Figure 1:**
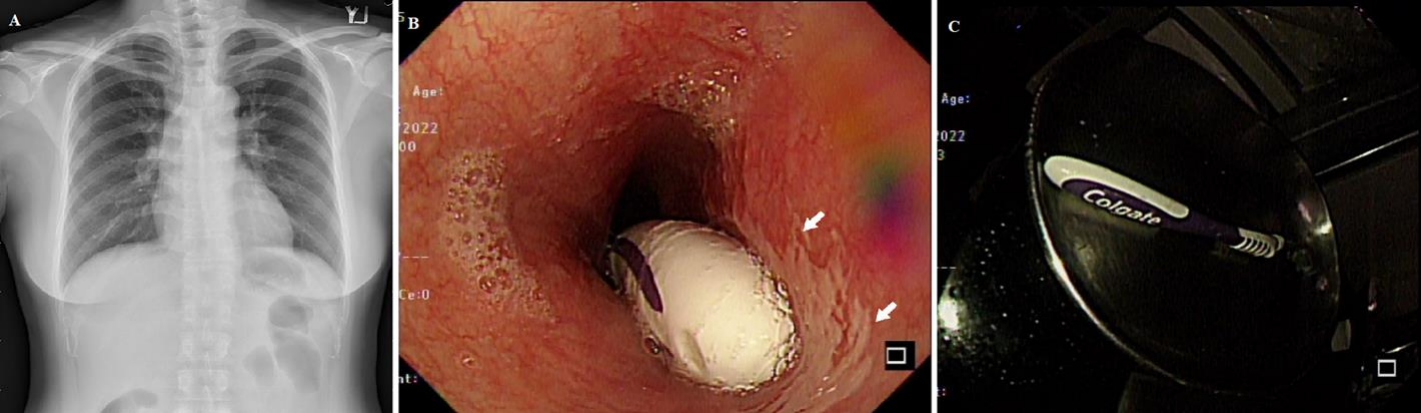
A) chest X-ray showing no evidence of toothbrush; B) toothbrush impacting at lower esophagus with ulcer formation; C) the 18cm impacting toothbrush was removed from the esophagus endoscopically

